# A CLCA regulatory protein present in the chemosensory cilia of olfactory sensory neurons induces a Ca^2+^-activated Cl^−^ current when transfected into HEK293

**DOI:** 10.1186/s12868-017-0379-7

**Published:** 2017-08-11

**Authors:** Casilda V. Mura, Ricardo Delgado, María Graciela Delgado, Diego Restrepo, Juan Bacigalupo

**Affiliations:** 1 Department of Biology, Faculty of Sciences, University of Chile, Las Palmeras 3425, Ñuñoa, 7800024 Santiago, Chile; 2 Department of Cell and Developmental Biology, Neuroscience Program and Rocky Mountain Taste and Smell Center, University of Colorado Anschutz Medical Campus, Aurora, CO 80045 USA

**Keywords:** CLCA, Ca^2+^-activated Cl^−^ current, Anoctamin channels, Olfactory cilia

## Abstract

**Background:**

CLCA is a family of metalloproteases that regulate Ca^2+^-activated Cl^−^ fluxes in epithelial tissues. In HEK293 cells, CLCA1 promotes membrane expression of an endogenous Anoctamin 1 (ANO1, also termed TMEM16A)-dependent Ca^2+^-activated Cl^−^ current. Motif architecture similarity with CLCA2, 3 and 4 suggested that they have similar functions. We previously detected the isoform CLCA4L in rat olfactory sensory neurons, where Anoctamin 2 is the principal chemotransduction Ca^2+^-activated Cl^−^ channel. We explored the possibility that this protein plays a role in odor transduction.

**Results:**

We cloned and expressed CLCA4L from rat olfactory epithelium in HEK293 cells. In the transfected HEK293 cells we measured a Cl^−^-selective Ca^2+^-activated current, blocked by niflumic acid, not present in the non-transfected cells. Thus, CLCA4L mimics the CLCA1 current on its ability to induce the ANO1-dependent Ca^2+^-activated Cl^−^ current endogenous to these cells. By immunocytochemistry, a CLCA protein, presumably CLCA4L, was detected in the cilia of olfactory sensory neurons co-expressing with ANO2.

**Conclusion:**

These findings suggests that a CLCA isoform, namely CLCA4L, expressed in OSN cilia, might have a regulatory function over the ANO2-dependent Ca^2+^-activated Cl^−^ channel involved in odor transduction.

**Electronic supplementary material:**

The online version of this article (doi:10.1186/s12868-017-0379-7) contains supplementary material, which is available to authorized users.

## Background

The CLCA1-4 proteins were previously considered to be Ca^2+^-activated Cl^−^ channels. However, it was later discovered that CLCAs were self-cleavage metalloproteases [1] found in different tissues, involved in Cl^−^ transport and mucus production [2]. Defective expression of CLCA genes can lead to different diseases such as asthma, cystic fibrosis and others [3, 4]. CLCA1 is the only CLCA family member that has been investigated in some detail so far. Heterologous expression of CLCA1 and exposure to extracellular CLCA1 induced an endogenous Anoctamin 1 (ANO1)-dependent Ca^2+^-activated Cl^−^ current in HEK293 cells [5]. This occurs because CLCA1 somehow promotes the incorporation and stabilization of the ANO1 channel into the plasma membrane [6]. CLCA1 shares its motif architecture with CLCA2, 3 and 4, suggesting that all these proteins are similarly processed and have an analogous function in different cell types [6]. By single-cell PCR in rat olfactory sensory neurons (OSNs), we previously found expression of the isoform CLCA4L as well as CLCA2 mRNAs, being CLCA4L mRNA much more abundant than the latter [7]. By immunoblotting of ciliary membranes as well as immunochemistry, we detected CLCA in the olfactory cilia, most likely CACL4L. The cilium is the cellular compartment where odor transduction takes place, a process in which the principal ionic current is carried by the Anoctamin 2 Ca^2+^-activated Cl^−^ channel (ANO2) [8]. To explore the possibility that CLCA4L could have a function in the cilia, we cloned it from rat olfactory epithelium, transfected it in HEK293 cells and characterized it electrophysiologically. This study reveals that CLCA4L can induce the HEK293 Ca^2+^-activated Cl^−^ current, thus mimicking CLCA1. Importantly, we also detected co-expression of CLCA with ANO2 in the cilia, as previously shown in toad olfactory sensory neurons [9]. The evidence provided is consistent with a possible role of CLCA in odor transduction, regulating ANO2.

## Methods

### Animals

Sprague–Dawley male rats were obtained from the Pontificia Universidad Católica de Chile. Animal care and experimental procedures were approved by the Bio-Ethical Committee of the Faculty of Sciences, University of Chile, according to the ethical rules the Biosafety Policy Manual of the Chilean National Fund for Science and Technology (FONDECYT). Rats were sacrificed with CO_2_ inhalation followed by decapitation.

### RNA preparation

Total mRNA from rat olfactory epithelium and other tissues such as liver and cerebellum were prepared by Trizolor RNeasy Plus, according to manufacturer’s instructions. Following extraction, the RNA was treated with RNase-free DNaseI to avoid any contamination with genomic DNA prior to use.

### Reverse transcription-PCR (RT-PCR)

To generate the first cDNA strand, total mRNA was transcribed with the reverse transcriptase Superscript III and oligo(dT). Primers were designed for maximal specificity using the DNA sequence encoding for rat CLCA4L (Gene ID: 499721, https://www.ncbi.nlm.nih.gov/gene/499721).

Equal amounts of total RNA from olfactory epithelium, liver and brain were used as templates in the reverse transcription reactions. The cDNA synthesis was carried out in the first step using total RNA primed with oligo(dT) in a 10 µL reaction volume containing dNTP mix to 0.5 mM final concentration. The reaction was heated at 65 °C during 5 min and placed on ice for at least 1 min. In the second step, a 10 µL mix containing 2 µL of 10× RT buffer, 1 µL of SuperScript III and 1 µL of RNase OUT, 4 µL of 25 mM MgCl_2_ and 2 µL of 0.1 M DTT, was added to the first step reaction. The 20 µL reverse transcription reaction was incubated first at 50 °C during 50 min and then inactivated at 85 °C for 5 min. Reverse transcriptase and RNA template negative controls were carried out in parallel for each of the analyzed samples.

### Full-length amplification and cDNA cloning

Full-length cDNA was obtained following several rounds of PCR using various sets of specific CLCA4L primers and *Pfu* DNA polymerase. Gene-specific amplification was performed with three independent PCR reactions using specific primers (Additional file 1: Figure S1, d), to produce three fragments (~1 kb long each). Fragment 5ʹ, of 1033 nucleotides, was prepared using forward primer Fw4EcoRI and reverse primer Rv2nw, the middle fragment, of 965 nucleotides, with primers Fw3nw and Rv3nw and the 5ʹend fragment, of 987 nucleotides, with primers Fw4nw and Rv4SmaI. The PCR protocol started with initial denaturation at 94 °C for 2 min, followed by 35 cycles at 94 °C during 30 s, 58 °C for 30 s, 72 °C for 1 min and a final extension step of 72 °C for 5 min. The PCR products were examined in RedGel stained 0.9% agarose gels to verify the expected sizes, and were gel purified with extraction from agarose using QIA quick gel extraction kit (Additional file 1: Figure S1, d).

To generate the full-length cDNA, a two-step PCR reaction was performed according to Shevchuck et al. [10] in which 20 µg of each of the three fragments were present, such that pairing and extension in the first cycle allowed amplification of the full-length cDNA in the subsequent cycles with primers Fw4EcoRI and Rv4SmaI.

The first step PCR protocol started with initial denaturation at 94 °C during 4 min, 20 cycles at 94 °C during 1 min, annealing at 72 °C with a 1 min decrease per each subsequent cycle, extension 72 °C for 1 min and a final extension step at 72 °C for 5 min. The second step PCR of 15 cycles started at 94 °C for 45 s, 52 °C 1 min, 72 °C 1 min and a final extension 72 °C for 5 min. The full-length cDNA fragment was digested with EcoR1 and SmaI, and cloned in pUC18 for amplification and sequencing. Correct size of the CLCA insert was verified by restriction mapping (Additional file 1: Figure S1, c) and the identity by homology search performed on the BLAST server (data not shown).

### Production of the expression vector coding for CLCA

The full-length cDNA cloned in pUC18 was released by digestion with Eco R1 and Sma I. Restriction enzyme-digested cDNA was directionally inserted downstream of the CMV promoter of pIRES2-EGFP for the expression of rat CLCA-enhanced green fluorescent (EGFP) fusion protein. Correct insertion was verified by restriction mapping. This construct containing the open reading frame of CLCA driven by the cytomegalovirus promoter was named pIRES2-CLCA4L and the region obtained by PCR was sequenced to confirm its identity.

### Cell line and culture conditions

HEK293 (ATCC^®^ CRL-1651™) cells were grown and maintained in Dulbecco’s modified Eagle medium (DMEM-F12) containing 2 mg/mL sodium bicarbonate, 100 µg/mL penicillin and 0.1 mg/L streptomycin. The culture medium was supplemented with 10% fetal bovine serum and 2 µM l-glutamine; cells were grown in a 5% CO_2_ atmosphere at 37 °C.

### Heterologous expression of CLCA4L

HEK293 cells were used to express the pIRES-CLCA4L coding plasmid and the pIRES2-EGFP original vector lacking the CLCA4L-insert, as a control. Cells were plated on 6-well plates at 5 × 10^5^ cells/well and transfected for 6 h with a mixture of 5 µL Lipofectamine 2000 and 2 µg of either pIRES-CLCA4L or the non-coding pIRES2-EGFP. Forty-eight hours after transfection, the cells were trypsinized and seeded into coverslips for electrophysiological studies. The HEK293 cells transfected with the plasmid pIRES2-CLCA4L were used within 3–9 days after transfection for patch-clamp characterization of the expressed CLCA4L.

### Immunofluorescence

Rat OSNs were obtained by mechanical dissociation of the olfactory mucosa in divalent cation-free solution (mM): 140 NaCl, 5 KCl, 10 Glucose, 10 HEPES, pH 7.4 at 37 °C. The cell suspension was gently passed several times through a 1 mL pipette tip, transferred to a Falcon tube and centrifuged at 300 rpm for 15 min. The supernatant was discarded and the pellet containing the cells was re-suspended in 8% paraformaldehyde (PFA), and left under slow shaking for 5 min at room temperature. The fixed cells were centrifuged (300 rpm for 15 min), the supernatant was discarded and the cells were re-suspended in phosphate-buffered saline (PBS); this procedure was repeated two more times. The cells were permeabilized by incubation in 0.3% Triton X100 in PBS at room temperature for 10 min. To block non-specific binding sites, the cells were incubated in 2% fetal bovine serum in PBS for 1 h. The primary antibodies, 1:200 mouse anti-CLCA [7] and 1:400 rabbit anti-ANO2 (Alomone Labs, Israel), were diluted in the same blocking solution and were incubated overnight at 4 °C under gentle shaking. The cells were centrifuged at 300 rpm for 10 min, the supernatant was discarded and the cells were washed twice in PBS. The cells were then incubated with secondary antibodies, mouse Alexa 488 and anti-rabbit Alexa 546 (Thermofisher, USA), prepared in PBS for 2 h in darkness with gentle shaking. The cells were centrifuged at 300 rpm for 5 min, the supernatant was discarded, the cells were resuspended and washed in PBS twice. Then, they were again centrifuged at 300 rpm for 10 min and incubated for 30 min in 50% Glycerol in PBS under gentle shaking. After this, a drop of the cell suspension was pipetted onto an untreated glass slide, and the cells were allowed to sink onto the coverslip and visualized with an inverted laser confocal microscope (Carl Zeiss LSM 710 Meta, Gottingen, Germany). A negative control was performed following the same protocol, but omitting incubation with the primary antibodies. The confocal images (0.1 µm slices) were captured using the same laser power and photo multiplier settings, and processed identically using Fiji (Image J) [11]. All images were projected axially and were deconvolved with Huygens Professional version 16.10 (Scientific Volume Imaging, The Netherlands). The final figure montage (Fig. 3) was performed with Adobe Photoshop, using identical values for contrast and brightness.

### Electrophysiology

HEK293 cells transfected with the pIRES2-EGFP-CLCA4L plasmid were viewed with an Olympus IX-70 fluorescence microscope (40× objective). Whole cell patch clamp was performed with an Axopatch-1D amplifier (Axon Instruments Inc., Novato CA, USA). A TL-1 Analog/Digital converter and pClamp 6 software (Axon Instruments Inc., Union City, CA, USA) were used for data acquisition (low-pass filtered at 2 kHz), data analysis. 1–2 MΩ resistance recording electrodes were made of glass capillaries pulled with a P-97 Microelectrode Puller (Sutter Instruments, Inc., Novato, CA, USA). To test for an effect of CLCA4L, the cells were voltage-clamped at −60 mV and whole cell currents were elicited by 900 ms voltage ramps from −60 to +60 mV. For a characterization of the CLCA4L-dependent currents, we used a tail currents protocol; This protocol consisted of jumping from a holding potential of −60–0 mV, switching after 300 ms to a sequence of 250 ms steps in increments of 15 mV, from −120 to +45 mV, and then returning back to −60 mV. Junction potentials were measured and subtracted when using asymmetrical solutions.

### Solutions

External solutions: Control Solution (in mM): 110 NMDG-Cl (N-Methyl-d-glucamin-Cl), 1 EGTA (ethylene glycol-bis(β-aminoethyl ether)-N,N,Nʹ,Nʹ-tetraacetic acid), 0.8 CaCl_2_, 10 HEPES, pH 7.0, 0.9 µM free Ca^2+^, calculated with Winmaxc 2 software (http://stanford.edu/cpatton/winmaxc2.html). Low-Ca^2+^ Solution: 110 NMDG-Cl, 1 BAPTA (1,2-bis(o-aminophenoxy)ethane-N,N,Nʹ,Nʹ-tetraacetic acid), 10 HEPES, pH 7.0, nominal Ca^2+^ 0.06 µM (Winmaxc 2 software). To block the Cl^−^ channel, 30 µM niflumic acid (NFA) was added to the Control Solution. Low Cl^−^ Solution: 100 NMDG-Acetate, 10 NMDG-Cl, 1 EGTA, 0.8 CaCl_2_, 10 HEPES, pH 7.0, 0.9 µM free Ca^2+^. In all these experiments the pipettes were filled with Control Solution.

In the ion-selectivity experiment, the external solutions were: 110 NaCl, 1 EGTA, 0.8 CaCl_2_, 10 HEPES, pH 7.0, 0.9 µM free Ca^2+^ and 110 Na-SCN, 1 EGTA, 0.8 CaCl_2_, 10 HEPES, pH 7.0, 0.9 µM free Ca^2+^. The pipette solution was: 110 NaCl, 1 EGTA, 0.8 CaCl_2_, 10 HEPES, pH 7.0, 0.9 µM free Ca^2+^.

The external solutions were replaced by bath perfusion. The solution containing niflumic acid was applied onto the cell with a puffer pipettes (~3 µm tip diameter) connected to a custom made picospritzer.

### Reagents

The molecular reagents TRIzolReagent, Oligo(dT)_12-18_, RNase free DNAase I, RNase OUT, RNase H, Super Script III, Lipofectamine 2000 and High DNA Mass Ladder were purchased from Invitrogen (San Diego CA, USA). RNeasy Plus Mini Kit, QIA quick PCR Purification, QIA quick gel extraction and QIAprep Spin Mini prep Kit from Qiagen (Valencia CA, USA). The *Pfu* DNA polymerase was from Promega (Madison WI, USA). T4 DNA ligase was from Thermo Scientific (Waltham, MA, USA). EZNA Endo-free Plasmid DNA mini kit from OMEGA Biotek (Norcross GA USA) was used to purify the plasmid for transfection. The vector pUC18 was from Stratagene, (Agilent Technologies Company, Santa Clara, CA, USA) and pIRES-GFP from BD Biosciences Clontech (San Jose, CA, USA). GelRed Nucleic Acid Stain was from Biotium, Hayward CA, USA) HEK293 cells were obtained from ATCC (American Type cell Collection Manassas, VA, USA). Dulbecco’s modified Eagle medium (DMEM-F12) and fetal bovine serum were from GIBCO (Invitrogen Corporation, San Diego CA, USA). Adobe Photoshop was from ADOBE Systems Mountain View, CA, USA). All other reagents were purchased from Sigma-Aldrich (St. Louis MO; USA).

## Results

### Cloning of the rat CLCA4L

We performed reverse transcriptase amplification of RNA isolated from olfactory epithelium. Using primers targeting the CLCA4L gene expressed in the rat nervous system allowed isolating three fragments corresponding to the full-length coding sequence of a CLCA gene present in the rat olfactory epithelium, which was named rOECLCA4L (Additional file 1: Figure S1). Sequence was verified by full-length sequencing of both strands of RT-PCR products from olfactory epithelium generated by the highly accurate *Pfu* DNA polymerase.

The sequence of the full-length cDNA (2798 bp) was analyzed by BLAST (http://www.ncbi.nlm.nih.gov/pubmed). The analysis revealed that this sequence presents 99% identity with the *Rattus norvegicus* CLCA4L, NM_001077356.2 and 88% identity with CLCA2 NM_001013202.2, in agreement with González-Silva et al. [7]. When compared with the CLCA4L, the rEOCLCA sequence shows three transitions, G to A at position 139, A to G at position 474 and A to G at position 2552, a transversion G to C at position 2383 and an insert of 13 nt at position 1018, which indicates that this is a new CLCA4 isoform.

**Fig. 1:**
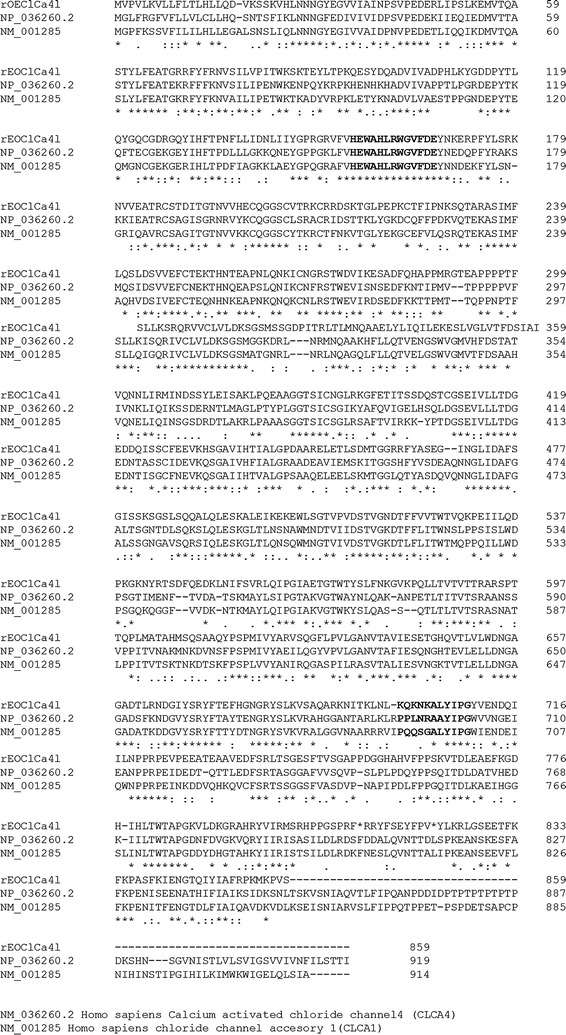
Amino acid sequence alignment of rOECLCA with hCLCA4, *Homo sapiens* Calcium activated chloride channel 4 NP_036260.2 and hCLCA1, *Homo sapiens* chloride channel accessory 1 NP_001276.2. Alignment was done by Clustal Omega Multiple Sequence alignment at EMBL-EBI (http://cbi.ac.uk/Tools/services/web/). An *asterisk* indicates a position with a single, fully conserved residue. *Colons* indicate conservation between groups of strongly similar properties as below, roughly equivalent to scoring >0.5 in the Gonnet PAM 250 matrix. *Periods* indicate conservation between groups of weakly similar properties as below, roughly equivalent to scoring <0.5 and >0 in the Gonnet PAM 250 matrix. The consensus catalytic motif HEXXHXXGXXDE at position 155 and the consensus cleavage site (position 695 for hCLCA1) are shown in *bold characters* [5]

The putative protein deduced from the coding sequence consists of 861 amino acids. The BLAST (http://www.ncbi.nlm.nih.gov/pubmed) analysis of the protein sequence showed 97% identity with *R. norvegicus* CLCA4-Like precursor, NP_001070824.1, 96% identity with *Homo sapiens* CLCA4 precursor, NP_036260.2, 84% identity with *Mus musculus* CLCA4, AAG23712.1 and 84% identity with *Mus musculus* CLCA4 precursor, NP_6318872. We carried out an amino acid sequence alignment of rat olfactory epithelium CLCA (rOECLCA4L) with hCLCA4 NP 036260.2 and hCLCA1 NP 001276.2 using CLUSTAL Omega (http://ebi.ac.uk/Tools/msa/clustalo/). The rOECLCA4L presents the common unique catalytic motif of zinc metalloproteases HEXXHXXGXXH (amino acids 150-162) and a consensus cleavage sequence similar to the wild type PQQSG-ALYIPG [6] (Fig. 1).

### CLCA4L-dependent Ca^2+^-activated Cl^−^ currents of transfected HEK293 cells

**Fig. 2:**
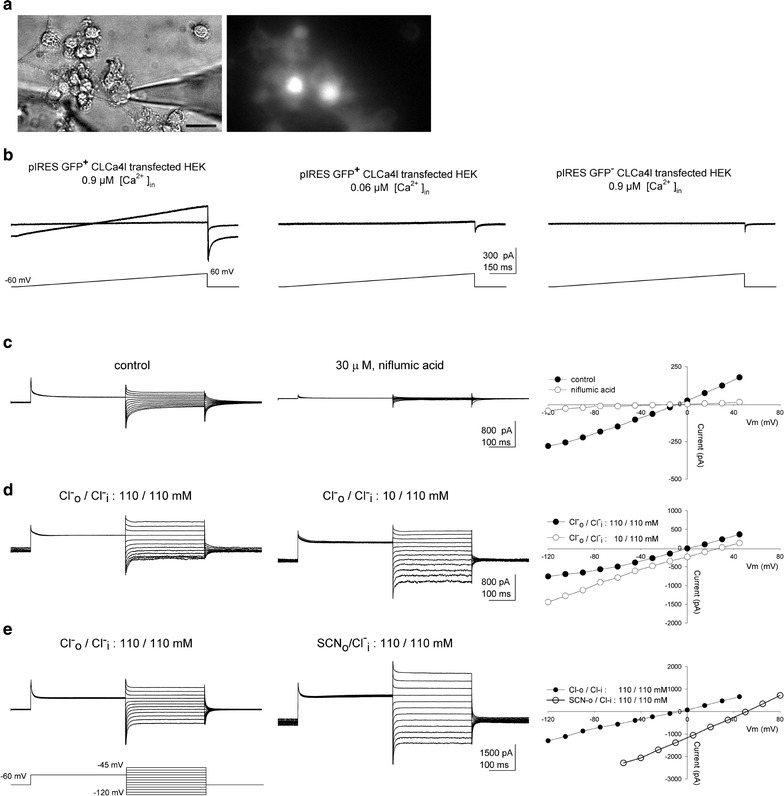
CLCA4L-mediated whole cell currents from HEK293 transfected with pIRES-2 EGFP-CLCA4L. **a** Cells under phase contrast; a patch clamp electrode is attached to one of the cells (*left*). Fluorescence image of the same field, with two cells displaying GFP-fluorescence, indicative of transfection (*right*). Calibration Bar: 10 µm. **b** *Left*, whole cell currents of a pIRES GFP^+^ CLCA4L-expressing cell under 0.9 µM Ca^2+^ internal solution, induced by voltage ramps in the absence (control) or presence of 30 µM external niflumic acid, and after washout; control and wash traces superpose (N = 6). *Center* a cell under 0.06 µM Ca^2+^ internal solution (N = 6). *Right* a pIRES GFP^+^ CLCA4L^−^ cell under 0.9 µM Ca^2+^ internal solution (N = 8); in all cases, ${\text{Cl}}_{\text{o}}^{ - } /{\text{Cl}}_{\text{I}}^{ - }$: 110/110. **c** *Left* whole cell currents of CLCA4L-HEK293 transfected cell induced by the tail current protocol depicted at the *bottom left*. *Center* under 30 µM niflumic acid (N = 6 both). *Right* I–V relationship. **d** *Left* currents under ${\text{Cl}}_{\text{o}}^{ - } /{\text{Cl}}_{\text{i}}^{ - }$: 110/110. *Center* ${\text{Cl}}_{\text{o}}^{ - } /{\text{Cl}}_{\text{I}}^{ - }$: 10/110. *Right* I–V relationship (N = 6, both). **e** *Left* currents under ${\text{Cl}}_{\text{o}}^{ - } /{\text{Cl}}_{\text{i}}^{ - }$: 110/110. *Center* under ${\text{SCN}}_{\text{o}}^{ - } /{\text{Cl}}_{\text{i}}^{ - }$ (110/110) (N = 6; see “Methods”). *Right* I–V relationship. Cells recorded in all experiments in the figure were representative

Figure 2a shows images of two HEK293 cells that were successfully transfected with the pIRES-2-GFP-CLCA4L construct, as revealed by their fluorescence, accompanied by non-transfected cells (not fluorescent). To test whether the transfected protein modified the membrane conductance, we recorded whole cell currents under voltage clamp. Voltage ramps from a holding potential of −60 mV to +60 mV elicited pronounced currents. Figure 2b illustrates the whole cell currents recorded from representative cells under symmetrical Cl^−^ and 0.9 µM free Ca^2+^ concentration in the internal solution, to keep Ca^2+^-gated channels open. A steady inward current was observed at −60 mV that rose linearly with the ramp, indicating a voltage-independent channel; the current reversed at 0 mV, as expected. We tested the Cl^−^ channel inhibitor niflumic acid (30 µM), added to the control solution and puffed with a double-barrel pipette at least 1 min before the onset of the ramp. The current was almost completely abolished by the inhibitor, an effect that was reversed after washing it out (Fig. 2c, left; N = 6). To examine whether the current was activated by Ca^2+^, we tested a CLCA4L-expressing cell dialyzed with 0.06 µM free Ca^2+^ solution. No current was observed at this low Ca^2+^ concentration, indicating that the current recorded with the ramp in the CLCA4L-expressing cells was Ca^2+^-activated (Fig. 2b, center; N = 6). To confirm that the Cl^−^ current was specifically due to the expression of CLCA4L in the HEK293 cells and not to endogenous presence of the protein, we examined cells transfected with the vector pIRES-GFP devoid of the CLCA4L. The Cl^−^ current was absent in these fluorescent cells under 0.9 µM internal Ca^2+^ (Fig. 2b, right; N = 8). These results show that the channel responsible for the Ca^2+^-activated Cl^−^ current was revealed only following the transfection of CLCA4L derived from rat olfactory epithelium.

Additional whole cell voltage clamp experiments were performed to further characterize the CLCA4L-induced Cl^−^ current in transfected HEK293 cells. We used a voltage protocol to measure tail currents under various conditions in order to build the respective current–voltage curves for determining the reversal potentials (see “Methods”). The currents evoked under control solutions in both sides (${\text{Cl}}_{\text{o}}^{ - } /{\text{Cl}}_{\text{i}}^{ - }$: 110/110, mM; Fig. 2c, left) were drastically inhibited by 30 µM niflumic acid (82%; 70 ± 14%; mean ± SD, p < 0.05; N = 6; Fig. 2c, center); the respective I–V relations are shown (Fig. 2c, right). To confirm that the currents were carried by Cl^−^, they were recorded under symmetrical control solution (Fig. 2d, left). Then, the Control solution was replaced by bath perfusion with a solution in which Cl^−^ was reduced to 10 and 100 mM Acetate replaced 100 mM Cl^−^. (Figure 2d, center). The current–voltage curve is shifted by 29 mV (31 ± 8 mV, mean ± SD, p < 0.01; N = 6; Fig. 2d, right), in agreement with the Acetate selectivity of Ca^2+^-activated Cl^−^ channels [12]. Finally, we explored the ionic selectivity of the of the Cl^−^ conductance by comparing the currents under symmetrical Cl^−^ solutions (110 mM Cl^−^; Fig. 2e, left) with those recorded after replacing external 110 mM Cl^−^ by 110 mM thiocyanate (Fig. 2e, center; N = 6). The reversal potential shifted by ~57 mV (53 ± 3 mV, mean ± SD, p < 0.01; Fig. 2e, right), in agreement with SCN^−^ selectivity of other Cl^−^ channels [12]. Altogether, the results confirm that the current recorded from HEK293 cells expressing the *clca4l* gene cloned from the olfactory epithelium is Ca^2+^-activated and carried by Cl^−^.

### Immunoreactivity for CLCA in the olfactory cilia

**Fig. 3:**
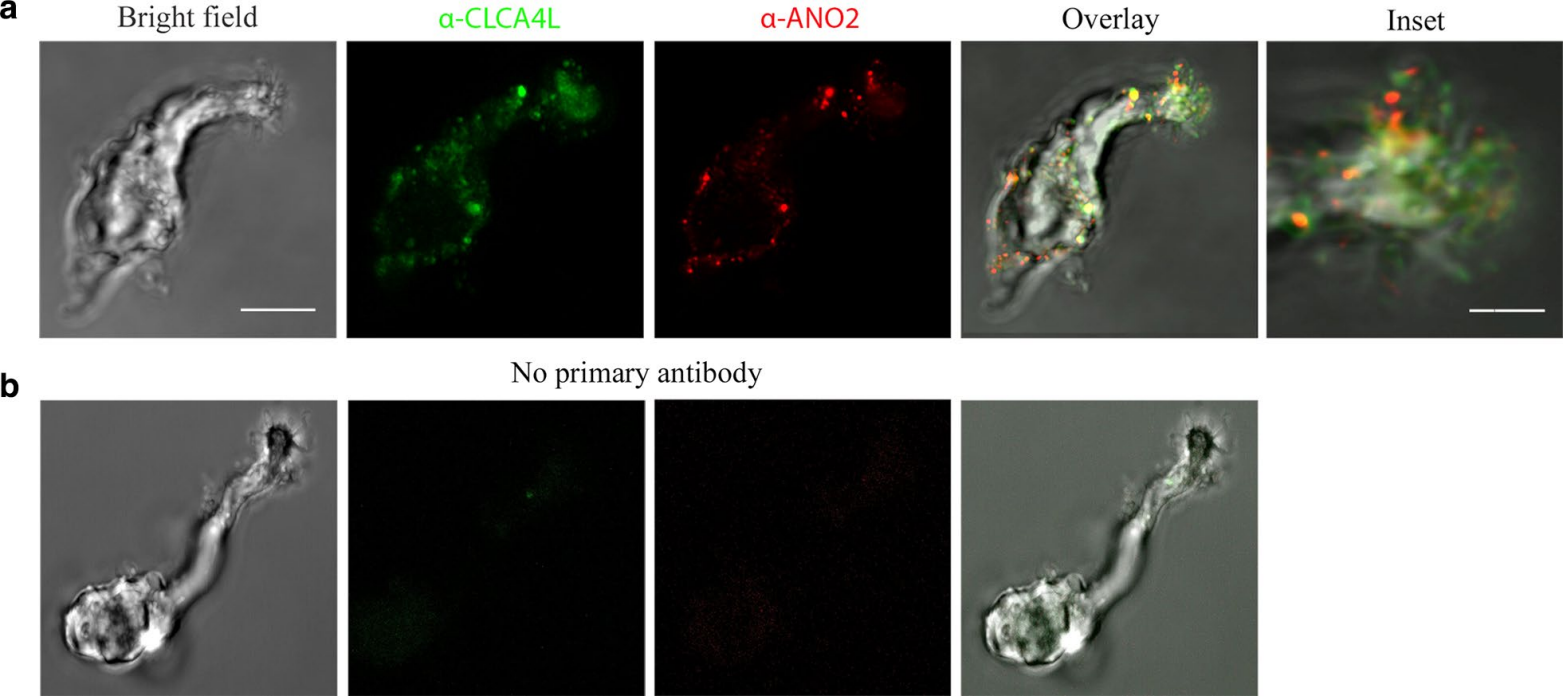
Co-expression of CLCA and ANO2 in olfactory cilia. **a** OSN under bright field, anti-CLCA immunofluorescence (*green*), anti-ANO2 immunofluorescence and overlay of three images; an *inset* of the apical region (*rectangle*) at higher magnification is shown on the *right* (N = 15); all fluorescent images were deconvolved. **b** Control in the absence of the primary antibodies. Neuron shown in bright field, immunofluorescence in the absence of anti-CLCA, immunofluorescence in absence anti-ANO2 and overlay of the three images (N = 12). *Bar*: 5 µm

We performed immunocytochemistry on isolated OSNs with an antibody against CLCA. Additionally, we used an anti-ANO2 antibody in order to compare the expression of both proteins in the cilia. Remarkably, the cilia displayed immunoreactivity to both, CLCA and ANO2 in all cells tested (Fig. 3a; N = 15). No immunoreactivity was detected in the absence of the primary antibody (Fig. 3b; N = 12). As mentioned above, the anti-CLCA antibody did not discriminate between CLCA4L and CLCA2, although according to the relative mRNA abundance, CLCA4L is most likely the protein in the cilia [7].

## Discussion

The odor transduction signaling cascade is triggered by a G-protein coupled odor receptor, which targets a cationic non-selective channel gated by cAMP (cyclic nucleotide-gated channel, CNG). The CNG channel provides Ca^2+^ ions for the activation of a Ca^2+^-activated Cl^−^ channel, ANO2 [8]. The ANO2-dependent current amplifies the depolarization initiated by the cationic current [13, 14]. A CLCA protein, presumably CLCA4L, was previously detected in olfactory cilia [7, 9]. We now document that this protein and ANO2 co-exist in these organelles. We additionally show that transfected CLCA4L mimics CLCA1 on inducing the ANO1-dependent Ca^2+^-activated Cl^−^ current of HEK293 cells, opening the interesting possibility that in olfactory cilia CLCA4L might play a regulatory role on ANO2.

Mammalian CLCA1-4 are metalloproteases with self-cleavage activity thought to modulate the activity of Ca^2+^-activated Cl^−^ channels. They are cleaved at a consensus site, giving raise to two fragments, one of them carrying the enzymatic activity [5, 15]. There is a close similarity between the amino acid sequence of the isoform that we cloned from the rat olfactory epithelium, rOECLCA4L, and the other three CLCAs [6], suggesting similar functions.

Of all the aforementioned CLCA proteins, only CLCA1 has been functionally investigated in some detail. In general, little is known about the role that such proteins play in modulating Cl^−^ fluxes in primary cells. In HEK293 cells, CACL1 somehow promoted the incorporation and stabilization in the plasma membrane of apparently pre-existing ANO1 channels when transfected or exogenously added to the culture medium, robustly incrementing its Cl^−^ conductance [5]. The mechanism by which these proteins are regulated has not been determined.

We found that CLCA4L cloned from the olfactory epithelium was able to induce a Ca^2+^-activated Cl^−^ current, highly selective for Cl^−^ and sensitive to the Cl^−^ channel blocker niflumic acid, when transfected in HEK293, possibly the ANO1-dependent current, resembling CLCA1. The endogenous expression of CLCA4L mRNA in OSNs [7], together with the presence of CLCA in their cilia co-expressing with ANO2, is consistent with a modulatory function over ANO2, with a possible role in odor transduction. Interestingly, CLCA4L had also been found in toad olfactory cilia [9], suggesting a widely conserved function in a wide range of animals.

## Conclusions

CLCA4L, a member of the CLCA metalloproteases that modulate Cl^−^ fluxes on a variety of epithelial cells, was also detected in mammalian olfactory epithelium. There, it is expressed in the odor transducing cilia of the olfactory sensory neurons, where the Ca^2+^-activated Cl^−^ channel ANO2 has a key role, suggesting a modulatory role over this channel.

## Additional file

**Additional file 1: Figure S1.** Construction of the full-length rEOCLCA4L cDNA. **a** Construction strategy to obtain the full-length cDNA by several rounds of PCR. First the cDNA (*dotted*) and PCR products, round 1, 2 and 3 respectively are depicted with black lines. **b** cDNA fragments corresponding to 3 fragments covering the full-length cDNA were obtained in PCR rounds and electrophoresed in a 1% agarose gel. *Lane 1*, shows fragment 5ʹ of 1033 nucleotides obtained with primers Fw4EcoRI and Rv2nw, *Lane 2* shows middle fragment of 965 nucleotides obtained with primers Fw3nw and Rv3nw, *Lane 3*, molecular weight marker (Gene Ruler 100 bp plus DNA Ladder, Fermentas, Germany), *Lane 4* shows fragment 5ʹend of 987 nucleotides with primers Fw4nw and Rv4SmaI. **c** Two samples of full-length cDNA 2786 nt, are shown in *lanes 1* and *3*. *Lane 2*: molecular weight marker. **d** CLCA4L primer sequences for PCR.

## Abbreviations

ANO: anoctamin channel family
CLCA: Ca^2+^-activated Cl^−^ channel regulator
CNG: cyclic nucleotide-gated channel
OSN: olfactory sensory neuron
