# Supplementary figures and images for: A CLCA regulatory protein present in the chemosensory cilia of olfactory sensory neurons induces a Ca2+-activated Cl− current when transfected into HEK293
Source: BMC Neurosci. 2017 Aug 11;18:61. doi: 10.1186/s12868-017-0379-7 (PMC5553735; doi:10.1186/s12868-017-0379-7)

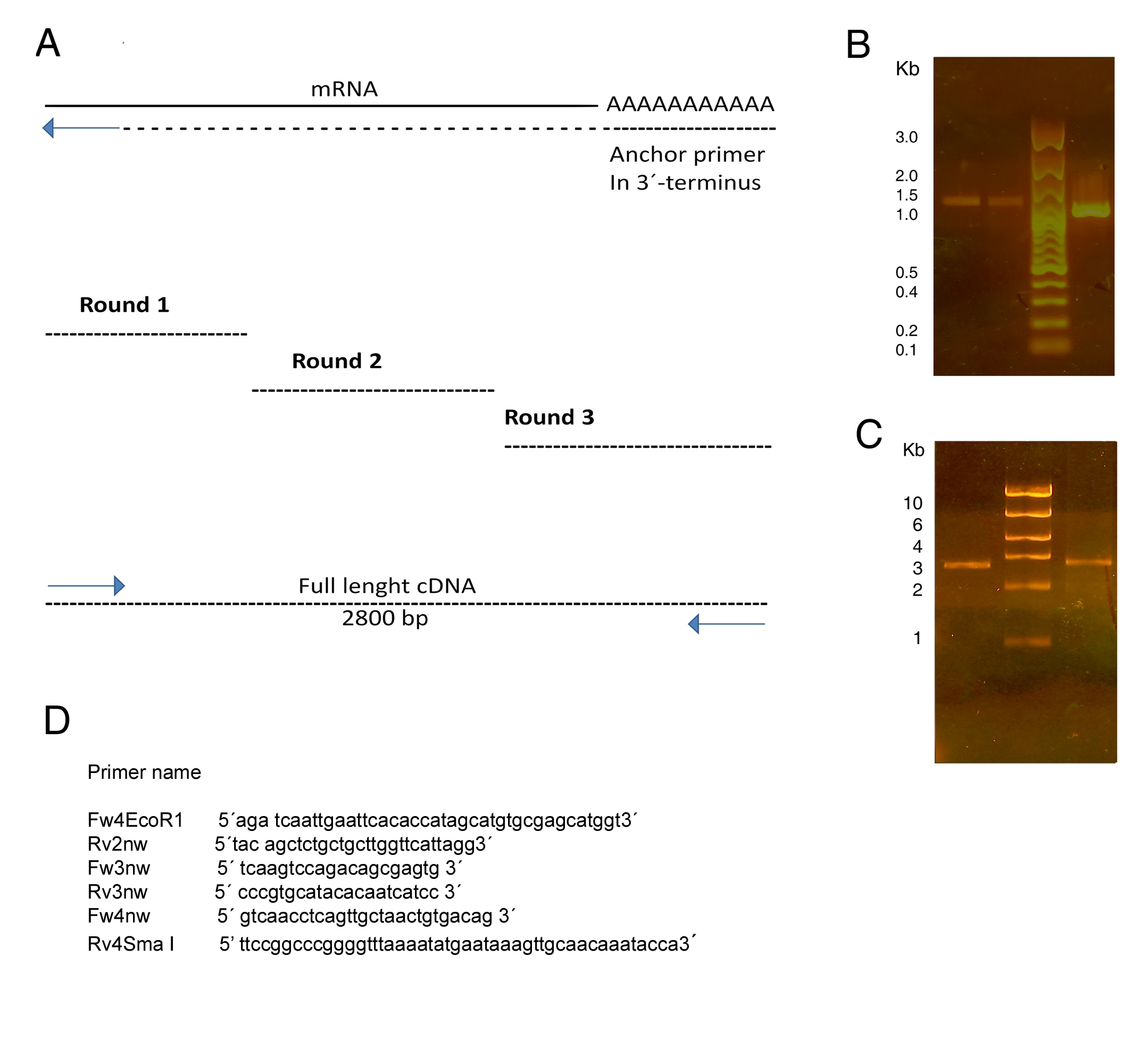

Supplement: Supplementary file 1 — Additional file 1: Figure S1. Construction of the full-length rEOCLCA4L cDNA. a Construction strategy to obtain the full-length cDNA by several rounds of PCR. First the cDNA (dotted) and PCR products, round 1, 2 and 3 respectively are depicted with black lines. b cDNA fragments corresponding to 3 fragments covering the full-length cDNA were obtained in PCR rounds and electrophoresed in a 1% agarose gel. Lane 1, shows fragment 5ʹ of 1033 nucleotides obtained with primers Fw4EcoRI and Rv2nw, Lane 2 shows middle fragment of 965 nucleotides obtained with primers Fw3nw and Rv3nw, Lane 3, molecular weight marker (Gene Ruler 100 bp plus DNA Ladder, Fermentas, Germany), Lane 4 shows fragment 5ʹend of 987 nucleotides with primers Fw4nw and Rv4SmaI. c Two samples of full-length cDNA 2786 nt, are shown in lanes 1 and 3. Lane 2: molecular weight marker. d CLCA4L primer sequences for PCR. [file 12868_2017_379_MOESM1_ESM.tif]
